# Research on International Cooperative Governance of the COVID-19

**DOI:** 10.3389/fpubh.2021.566499

**Published:** 2021-04-29

**Authors:** Xueyu Lin, Hualei Yang, Yuanyang Wu, Xiaodong Zheng, Lin Xie, Zheng Shen, Sen Hu

**Affiliations:** ^1^School of Public Administration, Zhongnan University of Economics and Law, Wuhan, China; ^2^School of Economics, Zhejiang Gongshang University, Hangzhou, China; ^3^Institute of Population and Labor Economics, The Chinese Academy of Social Science, Beijing, China; ^4^School of Economics and Management, Zhejiang A&F University, Hangzhou, China; ^5^School of Management Science and Engineering, Nanjing University of Information Science and Technology, Nanjing, China

**Keywords:** evolutionary game, novel coronavirus, COVID-19, international cooperative governance, health policy

## Abstract

Since the first case of the novel coronavirus (SARS-CoV-2) was reported in Wuhan, China, in December 2019, the coronavirus disease (COVID-19) has quickly spread to all the corners of the world. Amid the global public health threats posed by the COVID-19 pandemic, active cooperative governance has gradually emerged as the most powerful weapon against its spread. To facilitate international cooperation for pandemic governance, this paper applied the evolutionary game theory to analyze the factors influencing active cooperative governance and, based on the results, proposed a series of recommendations for promoting international cooperation. (1) leveraging the role of international organizations to reduce the cost of realizing the strategy of active cooperative governance, (2) promoting the international exchange of related experiences to lower the cost of active pandemic governance, (3) sustaining productive and daily activities during the pandemic in a classified and hierarchical manner to reduce the economic loss incurred by active pandemic governance, and (4) optimizing the incentive measures of international organizations to facilitate the selection of active cooperative governance. Finally, from the four aspects of resource management of pandemic treatment, supply management of living materials, population flow cooperation management, and governance fund cooperation management, this paper gives the path of international pandemic cooperative governance.

## Introduction

Since the first case of the novel coronavirus (SARS-CoV-2) was reported in Wuhan, China, in December 2019, the coronavirus disease (COVID-19) has quickly spread to all the corners of the world (1). In January 2020, the World Health Organization (WHO) declared the coronavirus outbreak a public health emergency of international concern (PHEIC) (2). Sometime around March 22, 2020, infections began to break out massively on a global scale, with a drastic spike reported in both confirmed cases and the death toll. As of March 11, 2021, a total of 118,584,961 confirmed cases and 2,630,190 deaths have been reported worldwide^1^. This global public health emergency has hitherto affected almost all countries and regions including Europe, North America, Asia, and Africa, the global landscape finds itself facing a grave crisis and projects a gloomy outlook.

Owing to its longevity and extensiveness, the COVID-19 outbreak has dealt a severe blow to the global socio-economic order (3). In 2020, in addition to China and Turkey, the economic growth of major economies in the world such as the United Kingdom, the United States, Germany, France, Italy, and Japan all was negative. On January 25, 2021, the Word Bank scaled down the global growth forecast by 0.2–3.2%. SARS-CoV-2, with its high potential of human-to-human transmission (4, 5), has wreaked economic havoc on global industries ranging from film and entertainment, catering, transportation and logistics, to tourism, retail, and export. The only effective remedy for this global socio-economic crisis is to contain the spread of COVID-19 as efficiently as possible.

A virus knows no borders or race. Although countries worldwide have rolled out countermeasures in response to the outbreak, their effectiveness has fallen short of expectations (6). In the face of the acuteness of this global public health crisis, Bruce Aylward, a senior advisor to the WHO, urged countries to foster information sharing and practice solidarity to wage a concerted battle against SARS-CoV-2. A single country's triumph will not bring the global pandemic to a close; the only way to declare an end to this global public health emergency once and for all is to ensure that every country can recover from its disruption (7). If SARS-CoV-2 is allowed to spread unchecked in vulnerable communities lacking access to testing equipment, ventilators, and medical supplies across poorer cities in Africa, Asia, and Latin America, it will linger there in the long run and reinvade other parts of the world, thus prolonging the public health crisis indefinitely (8, 9). Thus, in building a community with a shared future for mankind, countries must unite, join hands, and work in solidarity to defeat SARS-CoV-2 as soon as possible and minimize the loss caused by the pandemic (10–13). By searching the keyword “international cooperation to respond to the pandemic” in Web of Science, 277 related records were found, among which the top three topics were infectious diseases, public environmental occupational health, and international relations. There is no content directly related to international cooperation to control the pandemic situation or to promote the realization of cooperative governance. This topic has not been paid attention to in the current research.

Various strategies, based on lockdown, quarantine, increase medical and health resources, and international cooperation, to manage this pandemic have been extensively studied by numerous scholars (14–22). However, among all these studies, few have adopted the game theory as the theoretical and methodological underpinning for discussing the international cooperative governance against COVID-19. Active pandemic governance mainly refers to all countries in the world fighting COVID-19 through joint cooperation. As COVID-19 continues to spread to now also affect low resource countries who, under regular circumstances, have very limited capacity for intensive care, all countries hope that we will not repeat the mistakes of the past as seen with the HIV epidemic where life-saving drugs were only available in high resource countries, leaving impoverished nations with limited or no access to life-sustaining therapies. Therefore, it is necessary to govern through cooperation among countries, which is also advocated by the World Health Organization and expected by leaders of various countries, for example, President Xi Jinping attended the Extraordinary G20 Virtual Leaders' Summit and gave a speech titled “Working Together to Defeat the COVID-19 Outbreak” (23).

The reason why we use game theory to analyze lies in the tragedy of Commons in pandemic prevention and control. In the pandemic prevention and control, countries will face the dual choice of controlling the number of infected people and economic restart. If a country pays attention to the economic effect and ignores the increase in the number of infected people, it will choose to restart the economy as soon as possible. The close contact brought by the restart of economic activities will increase the number of infected people, thus aggravating the infection situation of the whole region, which leads to the conflict between individual interests and collective interests. In this case, we need to use game theory to analyze. Game theory is widely used in environmental cooperative governance. Examples of using game theory to study global social and economic problems such as *Greenhouse Gas Reduction Coalition and Its Stability Analysis*—*Based on the Perspective of Game Theory* (24), which examines the emission reduction actions of various countries from the perspective of game theory, and analyzes the possible cooperative emission reduction modes of various countries by using single alliance, Kyoto alliance and generalized Alliance respectively; *The Games of All Interest Groups Around the World in Carbon Emission Reduction and Some Discussions on China's Strategies* (25), which tries to use games theory to explain some contents between all interest groups in “Kyoto Protocol” and points out the causations of their contention; and *International Carbon Reduction Game with Low Carbon Development and China's Countermeasures* (26), which builds a game model to analyze the strategies of international carbon reduction parties at different stages.

In the current study, the application of game theory mainly focuses on non-cooperative game and cooperative game. A cooperative game is a game in which individuals cooperate to maximize the interests of the team, so as to promote the optimization of individual interests. The game theory focuses on the game behavior and strategy of the relevant stakeholders, which is suitable for the study of the cooperative relationship between countries. In the research object, game theory is aimed at different decision-making subjects, each subject represents their own interests, through the game behavior between different subjects to form an internal or external balance state. In the cooperative management of the pandemic situation, the game between governments is a repeated game process of random pairing and mutual learning, and its strategy adjustment process can be simulated by a replication dynamic mechanism. The evolutionary game analysis can reflect the behavior evolution path and stability strategy of governments in pandemic control, which has a certain reference significance for better carrying out pandemic control and reducing the losses caused by the pandemic. As far as research questions and content are concerned, few scholars have delved into issues such as how to enable and partake in international cooperative governance against COVID-19. Thus, the marginal contributions of this paper are two-fold. First, compared to previous studies (27–29), this paper enriches the research methodology on international cooperative governance against the COVID-19 outbreak by drawing on evolutionary game theory. Second, while numerous scholars have called upon international communities to collectively fight against COVID-19 (30, 31), studies on how to achieve international cooperation have been found wanting. To this end, the present study contributes to research content by exploring the enabling mechanisms and cooperative pathways at play based on an evolutionary game.

This paper is structured as follows: first, the evolutionary game model is employed to analyze the factors influencing the strategic choice of active cooperative governance, both with and without constraints. Second, it analyzes the ways to enable the strategic selection of active cooperative governance by regulating variables such as the cost of active pandemic governance, cost of active cooperative governance, the economic loss incurred by active pandemic governance, and incentives for active and passive pandemic governance. The enabling mechanisms of international cooperative governance are analyzed and examined from four aspects: reducing the cost of cooperation by leveraging the role of international organizations, reducing the cost of governance by sharing experiences in pandemic governance, reducing economic loss by sustaining production during the pandemic, and guiding active cooperative governance by optimizing incentives. Lastly, this paper discusses the ways to partake in cooperative governance based on four aspects: management of COVID-19 relief resources, management of daily supplies, cooperative management of population movement, and cooperative management of government funds.

## Methods

### Variable Description

As the outbreak of COVID-19, a country or region first encounters the health crisis caused by the spread of the virus, and the intervention measures, such as isolation to prevent the spread of the pandemic, will reduce the number of infected persons, control the spread of the pandemic, and achieve prevention and control gains; secondly, economic development can bring economic benefits to the country or region, and the pandemic prevention and control will affect the economic recovery progress of the country or region and affect economic benefits. At the same time, due to population mobility, infected people will flow between countries. If one country actively controls the pandemic and the other country responds negatively, infected people from countries that respond negatively will have negative externalities, which will affect the prevention and control effect of other countries. If the two countries actively respond, reach a cooperative alliance and jointly control the pandemic situation, it will bring common benefits of pandemic prevention and control, such as regional traffic recovery and smooth foreign trade.

Let *Ri*_1_ and *Ri*_2_ represent the individual benefit arising from the active pandemic governance undertaken separately by two affected countries, such as the national health brought by the control of pandemic, whereas *Rp*_1_ and *Rp*_2_ denote the public returns arising from the same, such as the positive externality brought by the prevention of infected people. *Rp* and *Rs*, respectively, stand for the public and shared returns gained when active pandemic governance is undertaken by both the affected countries, the public returns are economic benefits created by the restoration of public transport and trade in the region, the shared returns are the benefits of cooperation between the two countries; in most cases, *Rp* > *Rp*_1_ + *Rp*_2_. *Cp*_1_ and *Cp*_2_ represent the costs of active pandemic governance undertaken by both affected countries, such as the medical cost of treating infected patients within the country, whereas *Lp*_1_ and *Lp*_2_ indicate the losses incurred by the two affected countries because of the pandemic, such as the health loss caused by the death of infected patients. *Le*_1_ and *Le*_2_ denote the economic losses caused by active pandemic governance undertaken by both the affected countries, such as business stops and financial allocation to buy medical supplies. *Ce* is the cost incurred by the two affected countries realizing the strategy of active cooperative governance, such as the cost of negotiations, contracts, and political agenda between the two countries to achieve cooperation. θ denotes the externality coefficient between the affected countries. It is assumed that the negative externality coefficient of the pandemic equals its positive externality coefficient, where 0 < θ < 1. *E* is the reward conferred by a coalition on both players involved in active cooperative governance, for example, WHO provides financial assistance to regions or countries with active cooperation, while *F* is the punishment levied by the same on an affected country for passive governance, for example, WHO criticizes the United States for its negative response to the pandemic. When one of the two players partake in passive governance, the other will receive a subsidy, *Sf*, from the coalition for its active pandemic governance, such as the scientific research and material support provided by the World Health Organization to the country that actively responds. All the parameters above are positive values.

### Evolutionary Game Model

The model settings are mainly derived from the evolutionary game model of air pollution control developed by Gao et al. (32). Without the constraint of the coalition, whether an affected country undertakes active governance depends on the associated costs and returns. Similarly, whether or not it partakes in active cooperative governance is conditioned by the associated transaction costs, and shared and public returns. In a payoff matrix, when both affected countries opt for active governance, they will be subject not only to the individual returns, costs, and economic losses associated with active pandemic governance but also to the public and shared returns, arising from active cooperative governance, as well as the cost of realizing the strategy of active cooperative governance.

When both affected countries adopt the strategy of passive pandemic governance, their respective returns will be −*Lp*_1_ − θ*Lp*_2_ and −*Lp*_2_ − θ*Lp*_1_, respectively. When Affected Country 1 chooses active pandemic governance, and Affected Country 2 chooses a passive alternative, the former's returns will be *Ri*_1_ + *Rp*_1_ − *Cp*_1_ − *Le*_1_ − θ*Lp*_2_, and the latter's returns will be −*Lp*_2_ + θ*Lp*_2_. When their choices are swapped, the returns of Affected Countries 1 and 2 will be −*Lp*_1_ + θ*Lp*_1_ and *Ri*_2_ + *Rp*_2_ − *Cp*_2_ − *Le*_2_ − θ*Lp*_1_, respectively. When both countries choose active pandemic governance, their returns will be *Ri*_1_ + *Rp* + *Rs* − *Cp*_1_ − *Le*_1_ − *Ce* and *Ri*_2_ + *Rp* + *Rs* − *Cp*_2_ − *Le*_2_ − *Ce*, respectively. The payoff matrix of the two affected countries under different strategies is shown in Table 1.

**Table 1:** Payoff matrix between two affected countries.

	**Active governance by Affected Country 2**	**Passive governance by Affected Country 2**
Active governance by Affected Country 1	*Ri*_1_ + *Rp* + *Rs* − *Cp*_1_ − *Le*_1_ − *Ce*, *Ri*_2_ + *Rp* + *Rs* − *Cp*_2_ − *Le*_2_ − *Ce*	*Ri*_1_ + *Rp*_1_ − *Cp*_1_ − *Le*_1_ − θ*Lp*_2_, − *Lp*_2_ + θ*Lp*_2_
Passive governance by Affected Country 1	− *Lp*_1_ + θ*L,* *Ri*_2_ + *Rp*_2_ − *Cp*_2_ − *Le*_2_ − θ*Lp*_1_	− *Lp*_1_ − θ*Lp*_2_, − *Lp*_2_ − θ*Lp*_1_

Suppose the probabilities that the strategy of active pandemic governance is chosen by Affected Countries 1 and 2 equal *x* and *y*, respectively. Then, the probabilities of them selecting passive pandemic governance are defined as 1 − *x* and 1 − *y*, respectively.

The expected utilities of Affected Countries 1 and 2 when both choose active pandemic governance are defined as follows:

(1) $$\begin{array}{l} \{\begin{array}{l} u_{11}=y\left(Ri_{1}+Rp+Rs-Cp_{1}-Le_{1}-Ce\right)\\ \;+\left(1-y\right)\left(Ri_{1}+Rp_{1}-Cp_{1}-Le_{1}-\theta Lp_{2}\right)\\ u_{21}=x\left(Ri_{2}+Rp+Rs-Cp_{2}-Le_{2}-Ce\right)\\ \;+\left(1-x\right)\left(Ri_{2}+Rp_{2}-Cp_{2}-Le_{2}-\theta Lp_{1}\right) \end{array} \end{array}$$

The expected utilities of Affected Countries 1 and 2 when both choose passive pandemic governance are expressed as follows:

(2) $$\begin{array}{l} \{\begin{array}{l} u_{12}=y\left(-Lp_{1}+\theta Lp_{1}\right)+\left(1-y\right)\left(-Lp_{1}-\theta Lp_{2}\right)\\ u_{22}=x\left(-Lp_{2}+\theta Lp_{2}\right)+\left(1-x\right)\left(-Lp_{2}-\theta Lp_{1}\right) \end{array} \end{array}$$

The average expected utilities of Affected Countries 1 and 2 when active and passive pandemic governance, respectively, are chosen are as follows:

(3) $$\begin{array}{l} \{\begin{array}{l} {\tilde{u}}_{1}=xu_{11}+\left(1-x\right)u_{12}\\ {\tilde{u}}_{2}=yu_{21}+\left(1-y\right)u_{21} \end{array} \end{array}$$

To obtain the evolutionary equilibrium strategy of each affected country, it is important to first define the replicator dynamics equations for Affected Countries 1 and 2, and then let them be 0. With that as a necessary condition for the evolutionary equilibrium strategy, the equilibrium must also be solved according to Friedman's method. The replicator dynamics equations are defined as follows:

(4) $$\begin{array}{l} \{\begin{array}{c} F\left(x\right)=\frac{dx}{dt}==x\left(u_{11}-{\tilde{u}}_{1}\right)=x(1-x)(u_{11}-u_{12})\\ F\left(y\right)=\frac{dy}{dt}=y(u_{21}-{\tilde{u}}_{2})=y(1-y)(u_{21}-u_{22}) \end{array} \end{array}$$

**Figure 1:**
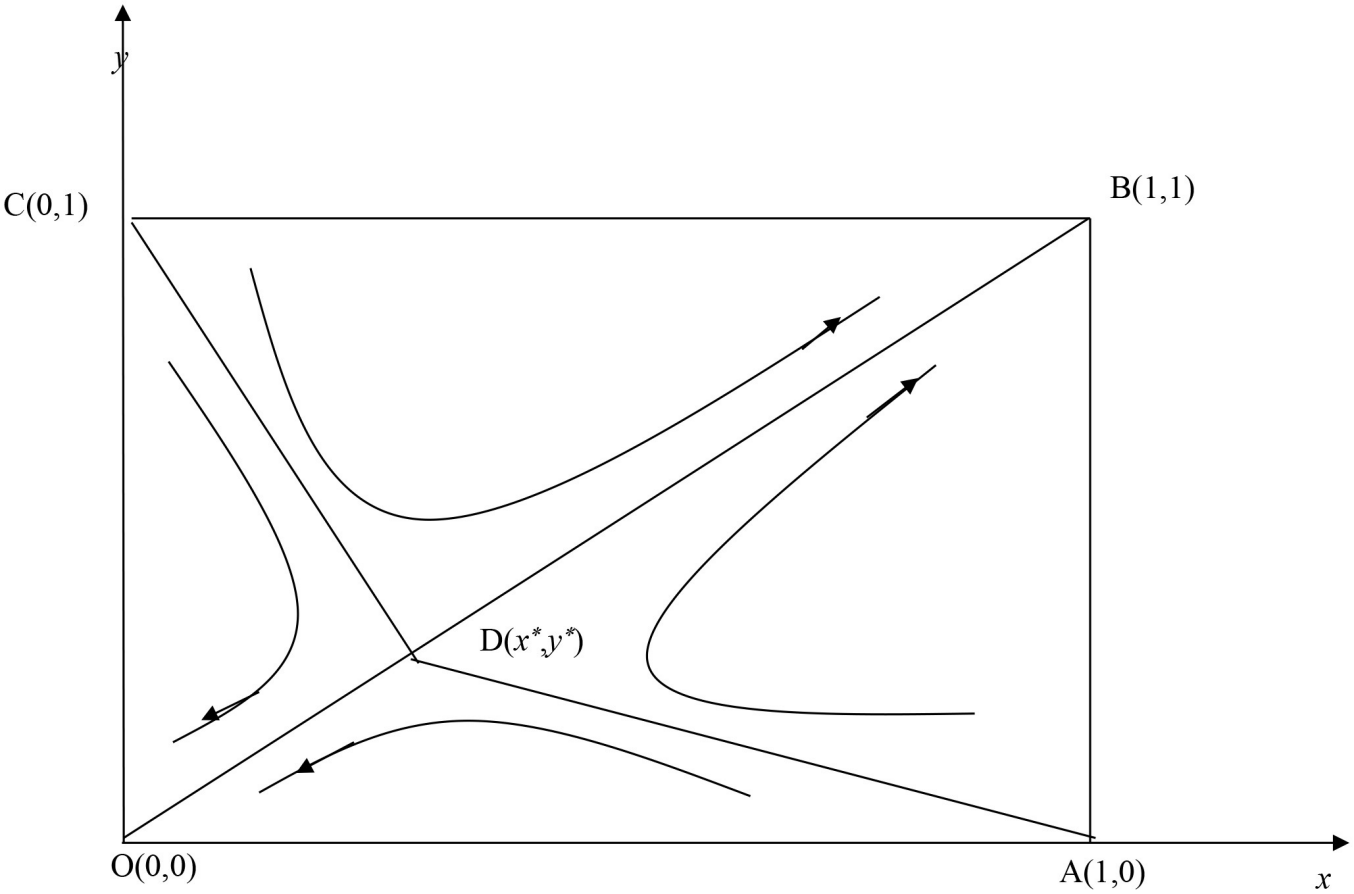
Evolutionary game phase diagram of cooperative governance.

Let *F*(*x*) and *F*(*y*) be 0. Five strategy equilibrium points for Affected Countries 1 and 2 are obtained: O (0, 0), C (0, 1), A (1, 0), B (1, 1), and D (x^*^, y^*^), where x^*^ = $\frac{-Ri_{2}-Rp_{2}-Lp_{2}+Cp_{2}+Le_{2}}{Rp+Rs-Rp_{2}-\theta Lp_{2}-Ce}$, $y^{*}=\frac{-Ri_{1}-Rp_{1}-Lp_{1}+Cp_{1}+Le_{1}}{Rp+Rs-Rp_{1}-\theta Lp_{1}-Ce}$. The specifics are shown in Figure 1.

## Results

### Basic Model Analysis

According to Friedman's methods, this study identifies (0, 0) and (1, 1) as the evolutionarily stable strategies for the two affected countries. The corresponding strategies are for both countries to choose passive and active pandemic governance, respectively, with (x^*^, y^*^) as the saddle point. When the probability of active governance being initially adopted by both Affected Countries 1 and 2, (*x*_0_, *y*_0_), falls within ABCD, the two countries will lean toward the strategy profile of active cooperative governance over time. In other words, the system will converge to (1, 1). However, when the said probability falls within AOCD, the two countries will lean toward passive non-cooperative governance, and the system will converge to (0, 0). The details are mentioned in Figure 1. The surface area of ABCD should be expanded to increase the probability of the system converging to the state of active cooperative governance along BD. The surface area of ABCD is defined as follows:

(5) $$\begin{array}{l} S_{ABCD}=1-\frac{x^{*}+y^{*}}{2} \end{array}$$

Substitute the values of x^*^ and y^*^ into *S*_*ABCD*_ to obtain the following equation:

(6) $$\begin{array}{l} S_{ABCD}=1+\frac{1}{2}\frac{Ri_{2}+Rp_{2}+Lp_{2}-Cp_{2}-Le_{2}}{Rp+Rs-Rp_{2}-\theta Lp_{2}-Ce}\\ \;+\frac{1}{2}\frac{Ri_{1}+Rp_{1}+Lp_{1}-Cp_{1}-Le_{1}}{Rp+Rs-Rp_{1}-\theta Lp_{1}-Ce} \end{array}$$

To enable each affected country to converge to the state of active cooperative governance, this study first observed the effects of changes in the following parameters on *S*_*ABCD*_: costs of active pandemic governance (*Cp*_1_ and *Cp*_2_), economic losses caused by active pandemic governance (*Le*_1_ and *Le*_2_), and the cost of realizing the strategy of active cooperative governance (*Ce*). The partial derivatives of *Cp*_*k*_, *Le*_*k*_, and *Ce* are computed with respect to *S*_*ABCD*_. Considering that the above parameters are all above 0, *Rp* + *Rs* > *Rp*_*k*_ − θ*Lp*_*k*_ − *Ce*, and *Ri*_*k*_ + *Rp*_*k*_ + *Lp*_*k*_ < *Cp*_*k*_ + *Le*_*k*_, the following can be obtained:

(7) $$\begin{array}{l} \{\begin{array}{lll} \frac{\partial S_{ABCD}}{\partial Cp_{k}} & = & \frac{-1}{2\left(Rp+Rs-Rp_{k}-\theta Lp_{k}-Ce\right)}<0\\ \frac{\partial S_{ABCD}}{\partial Le_{k}} & = & \frac{-1}{2\left(Rp+Rs-Rp_{k}-\theta Lp_{k}-Ce\right)}<0\\ \frac{\partial S_{ABCD}}{\partial Ce} & = & \sum_{k=1}^{2}\frac{Ri_{k}+Rp_{k}+Lp_{k}-Cp_{k}-Le_{k}}{{2\left(Rp+Rs-Rp_{k}-\theta Lp_{k}-Ce\right)}^{2}}<0 \end{array} \end{array}$$

This implies that, if the costs of active pandemic governance, the economic losses caused by such governance, and the cost of realizing the strategy of active cooperative governance are lower, the affected countries are more likely to choose the strategy of active cooperative governance over time.

In reality, first of all, in terms of the cost of controlling the pandemic, due to the differences in the economic level between countries and the ability of national financial investment, developed countries can spend more manpower and material resources to control the pandemic, while poor countries cannot afford the huge investment in pandemic prevention materials; secondly, in terms of the capacity of bearing economic loss, in order to reduce the death and spread of patients, developed countries tend to take longer-term measures of trade stop, blockade and isolation, while poor areas cannot afford long-term economic losses. Finally, in terms of the cost of cooperation strategy, if the two countries have a similar political and economic background and previous cooperation foundation, the resistance of cooperation between the two countries will be greatly reduced, thus contributing to the realization of cooperation. However, if there are huge economic differences and national contradictions, the increase of cooperation costs will hinder the realization of cooperative governance. Therefore, the lower the cost of controlling the pandemic, the smaller the economic loss and the lower the cost of achieving the cooperation strategy are conducive to the realization of the cooperation strategy.

Furthermore, the effects of the following parameters on *S*_*ABCD*_ are also observed: individual returns of active pandemic governance (*Ri*_1_ and *Ri*_2_), public returns of active cooperative governance (*Rp*), shared returns of active cooperative governance (*Rs*), and losses incurred by the pandemic (*Lp*_1_ and *Lp*_2_). The partial derivatives of *Ri*_*k*_, *Rp*, *Rs*, and *Lp*_*k*_ are obtained with respect to *S*_*ABCD*_. Considering that θ > 0, *Rp* + *Rs* > *Rp*_*k*_ − θ*Lp*_*k*_ − *Ce*, and *Ri*_*k*_ + *Rp*_*k*_ + *Lp*_*k*_ < *Cp*_*k*_ + *Le*_*k*_, the following can be obtained:

(8) $$\begin{array}{l} \{\begin{array}{lll} \frac{\partial S_{ABCD}}{\partial Ri_{k}} & = & \frac{1}{2\left(Rp+Rs-Rp_{k}-\theta Lp_{k}-Ce\right)}>0\\ \frac{\partial S_{ABCD}}{\partial Rp} & = & \sum_{k=1}^{2}\frac{Cp_{k}+Le_{k}-Ri_{k}+Rp_{k}+Lp_{k}}{{2\left(Rp+Rs-Rp_{k}-\theta Lp_{k}-Ce\right)}^{2}}>0\\ \frac{\partial S_{ABCD}}{\partial Rs} & = & \sum_{k=1}^{2}\frac{Cp_{k}+Le_{k}-Ri_{k}+Rp_{k}+Lp_{k}}{{2\left(Rp+Rs-Rp_{k}-\theta Lp_{k}-Ce\right)}^{2}}>0\\ \frac{\partial S_{ABCD}}{\partial Lp_{k}}\\ & = & \sum_{k=1}^{2}\left[\frac{\theta \left(Ri_{k}+Rp_{k}+Lp_{k}-Cp_{k}-Le_{k}\right)+Rp+Rs-Rp_{k}-\theta Lp_{k}-Ce}{{2\left(Rp+Rs-Rp_{k}-\theta Lp_{k}-Ce\right)}^{2}}\right]\;>0 \end{array} \end{array}$$

This implies that, if the individual returns of active pandemic governance, the public and shared returns of active cooperative governance, and the losses incurred by the pandemic are higher, the affected countries are more likely to choose the strategy of active cooperative governance over time.

Specifically, in reality, first of all, in the area of self-earnings, controlling the pandemic situation and reducing the number of infected persons will bring their own benefits to the national health, the credibility of the government, and the enhancement of the international image, which will promote the countries to actively respond to COVID-19. Secondly, active governance will reduce the number of domestic infections and reduce the number of domestic infections that will affect other countries' prevention of the pandemic. The prevention in own country can bring positive externalities to the prevention of other countries. At the same time, the cooperative governance will bring the partners the common benefits of the two countries' trade recovery. The improvement of public benefits and common benefits will promote cooperation. Finally, in terms of the harm of the pandemic, the more serious the economic and social harm caused by the pandemic, the more difficult it is for a single country to deal with it, and the need for other countries to cooperate, the easier the cooperation strategy will be achieved. Therefore, the greater the individual benefits, public benefits, common benefits, and the harm of the pandemic, the easier the cooperative governance strategy will be achieved.

### Extended Analysis

The above analysis is set against an unconstrained background. However, in reality, the affected countries are under constraints imposed by a variety of international organizations currently in force, including the United Nations (UN), WHO, World Trade Organization (WTO), IMF, International Labor Organization, and Food and Agriculture Organization (33, 34). To some degree, these international coalitions are analogous to a coalition government, which can reward or punish countries for active or passive pandemic governance. The WTO, for example, punishes countries for passive pandemic governance by imposing bans or tariff hikes on their import and export, while rewarding their active counterparts by lowering the import and export tariffs. Compared with the above strategy game which was without constraints, the coalition's punishment to countries for passive pandemic governance (*F*), subsidy for active pandemic governance (*Sf*), and reward for forming an alliance in active cooperative governance (*E*), are now added to the payoff matrix shown in Table 1. The revised payoff matrix under constraints is shown in Table 2.

**Table 2:** Payoff matrix between two affected countries under constraints.

	**Active governance by Affected Country 2**	**Passive governance by Affected Country 2**
Active governance by Affected Country 1	*Ri*_1_ + *Rp* + *Rs* − *Cp*_1_ − *Le*_1_ − *Ce* + *E*, *Ri*_2_ + *Rp* + *Rs* − *Cp*_2_ − *Le*_2_ − *Ce* + *E*	*Ri*_1_ + *Rp*_1_ − *Cp*_1_ − *Le*_1_ − θ*Lp*_2_ + *Sf*, −*Lp*_2_ + θ*Lp*_2_ − *F*
Passive governance by Affected Country 1	−*Lp*_1_ + θ*Lp*_1_ − *F*, *Ri*_2_ + *Rp*_2_ − *Cp*_2_ − *Le*_2_ − θ*Lp*_1_ + *Sf*	−*Lp*_1_ − θ*Lp*_2_ − *F*, −*Lp*_2_ − θ*Lp*_1_ − *F*

First, this study defines the system of replicator dynamics equations and lets them be 0. Once again, five strategy equilibrium points for the two affected countries can be obtained: O (0, 0), C (0, 1), A (1, 0), B (1, 1), and D (x^*^, y^*^), where $x^{*}=\frac{-Ri_{2}-Rp_{2}-Lp_{2}-F-Sf+Cp_{2}+Le_{2}}{Rp+Rs+E-Rp_{2}-\theta Lp_{2}-Ce-Sf}$ and $y^{*}=\frac{-Ri_{1}-Rp_{1}-Lp_{1}-F-Sf+Cp_{1}+Le_{1}}{Rp+Rs+E-Rp_{1}-\theta Lp_{1}-Ce-Sf}$.

Similarly, according to Friedman's method, the strategy equilibrium points (0, 0) and (1, 1) are identified as evolutionarily stable strategies. When the probability of active governance being initially adopted by both Affected Countries 1 and 2, (*x*_0_, *y*_0_), falls within ABCD, the countries will lean toward the strategy of active cooperative governance over time. In other words, the system will converge to B (1, 1). However, if the probability falls within AOCD, the countries will lean toward passive non-cooperative governance, and the system will converge to O (0, 0). If the probability of the affected countries choosing the strategy of active cooperative governance is to be increased over time, it is necessary that saddle point D is moved to the origin 0, in order to expand the surface area of ABCD. Considering that the surface area of ABCD is defined as $S_{ABCD}=1-\left(x^{*}+y^{*}\right)/2$, and the values of x^*^ and y^*^ are substituted into *S*_*ABCD*_, the following can be obtained:

(9) $$\begin{array}{l} S_{ABCD}=1+\frac{1}{2}(\frac{Ri_{2}+Rp_{2}+Lp_{2}+F+Sf-Cp_{2}-Le_{2}}{Rp+Rs+E-Rp_{2}-\theta Lp_{2}-Ce-Sf}\\ \;+\frac{Ri_{1}+Rp_{1}+Lp_{1}+F+Sf-Cp_{1}-Le_{1}}{Rp+Rs+E-Rp_{1}-\theta Lp_{1}-Ce-Sf}) \end{array}$$

The previous section has already probed the effects of the following parameters on *S*_*ABCD*_: costs of active pandemic governance (*Cp*_1_ and *Cp*_2_), economic losses caused by active pandemic governance (*Le*_1_ and *Le*_2_), the cost of realizing the strategy of active cooperative governance (*Ce*), individual returns of active pandemic governance (*Ri*_1_ and *Ri*_2_), public and shared returns of active cooperative governance (*Rp* and *Rs*), and losses incurred by the pandemic (*Lp*_1_ and *Lp*_2_).

In this section, the analytical focus shifts to the effects of the following parameters on *S*_*ABCD*_: coalition government's punishment for passive pandemic governance (*F*), subsidy for active pandemic governance (*Sf*), and reward for forming an alliance of active cooperative governance (*E*). The partial derivatives of *E*, *F*, and *Sf* are computed with respect to *S*_*ABCD*_. Considering that *Cp*_*k*_ + *Le*_*k*_ > *Ri*_*k*_ + *Rp*_*k*_ + *Lp*_*k*_ + *F* + *Sf*, *Rp* + *Rs* + *E* > *Rp*_*k*_ + θ*Lp*_*k*_ + *Ce* + *Sf*, and *Rp* + *Rs* > *Cp*_*k*_ + *Le*_*k*_ + *Ce*, the following can be obtained:

(10) $$\begin{array}{l} \{\begin{array}{lll} \frac{\partial S_{ABCD}}{\partial E} & = & \sum_{k=1}^{2}\frac{Cp_{k}+Le_{k}-Ri_{k}-Rp_{k}-Lp_{k}-F-Sf}{{2\left(Rp+Rs+E-Rp_{k}-\theta Lp_{k}-Ce-Sf\right)}^{2}}>0\\ \frac{\partial S_{ABCD}}{\partial F} & = & \sum_{k=1}^{2}\frac{1}{2\left(Rp+Rs+E-Rp_{k}-\theta Lp_{k}-Ce-Sf\right)}>0\\ \frac{\partial S_{ABCD}}{\partial Sf} & = & \sum_{k=1}^{2}\frac{Ri_{k}+\left(1-\theta \right)Lp_{k}+E+F+Rp+Rs-Cp_{k}-Le_{k}-Ce}{{2\left(Rp+Rs+E-Rp_{k}-\theta Lp_{k}-Ce-Sf\right)}^{2}}>0 \end{array} \end{array}$$

This means that if the joint organization punishes the countries that choose not to actively control the pandemic, subsidizes the countries that choose to actively control the pandemic, and rewards the alliance for actively cooperate to governance the pandemic, over time, each country will be more likely to choose the strategy of active cooperative governance. Specifically speaking, first, the punishment for the countries who negatively respond to the pandemic will promote the realization of cooperation, such as the World Health Organization's public criticism of the negative attitude of the United States in the early stage of the pandemic; second, subsidies will be given to the countries with active governance, and the financial assistance, scientific research and material assistance provided by the World Health Organization to the poor areas will help them control the pandemic. Finally, on the reward for the governance alliance, the WHO thanks the EU leaders for their efforts in uniting the world to fight the pandemic and providing the EU with materials for defeating the pandemic. Therefore, punishing the countries that negatively respond, subsidizing the countries that positively governance and rewarding the cooperative governance alliance are conducive to the achievement of cooperative strategies.

Summarizing the above, three conclusions can be drawn. First, if the costs of active pandemic governance, the economic losses incurred by such governance, and the cost of enabling the strategic choice of active cooperative governance are lower, the affected countries are more likely to choose active cooperative governance over time. Second, if the individual returns of active pandemic governance, the public and shared returns of active cooperative governance, and the socio-economic impacts of the pandemic are higher, the affected countries are more likely to adopt active cooperative governance over time. Third, if the coalition punishes countries for passive pandemic governance, subsidizes countries for active pandemic governance, and rewards alliances of active cooperative governance, the affected countries are more likely to opt for active cooperative governance over time. Additionally, the strategic continuity of active cooperative governance is determined by the magnitude of its returns and the constraints imposed by the coalition.

## Discussion

### Recommendations for Enabling Cooperative Governance

This study adopts the evolutionary game model to analyze factors influencing the strategic choice of active cooperative governance, along with the ways to enable such a choice. The individual returns of active pandemic governance, public and shared returns of active cooperative governance, and the socio-economic impacts of the pandemic are some of the more objective variables. Thus, to enable the strategic selection of active cooperative governance, efforts should be made to regulate the costs of active pandemic and active cooperative governance, the economic loss incurred by active pandemic governance, and the incentives for active and passive pandemic governance. The detailed measures are as follows: (1) leveraging the role of international organizations to reduce the cost of adopting active cooperative governance, (2) promoting the international exchange of related experiences to lower the cost of active pandemic governance, (3) sustaining productive and daily activities during the pandemic in a classified and hierarchical manner to reduce the economic loss incurred by active pandemic governance, and (4) optimizing the incentive measures of international organizations to guide countries adopt the strategy of active cooperative governance.

To reduce the cost of strategic selection, the UN—as the most authoritative of comprehensive international organizations—approved a draft resolution titled “Global Solidarity to Fight the Coronavirus Disease 2019 (COVID-19),” introduced by six countries including Singapore, Ghana, and Indonesia, during the UN General Assembly. The resolution was co-sponsored by over 180 countries. The General Assembly's call for global solidarity and concerted efforts against the pandemic marked an important step in advancing international cooperation. The approval of the draft resolution saved the costs of negotiation and contract administration and lowered the threshold and resistance against international cooperation.

To reduce the cost of governance, the mortality rate of COVID-19 patients approximated to 4% in China and exceeded 6% in other regions. It is necessary for the hardest-hit areas such as Europe to draw on China's therapeutic regimens for a higher recovery rate. China has shared its experience and practices with over 10 countries including France, Portugal, and Denmark with regard to virological characteristics, anti-pandemic philosophy, and the latest research achievements in pathology. It also imparted to Europe information that is highly instrumental in clinical treatments, including information on Chinese medicine and many other clinical regimens, as well as the recommended dosage, contraindications, and efficacy of an antimalarial drug and other agents.

To reduce economic losses, it is important to ensure the smooth and continued operations of the global economy and trade. On the one hand, excessive draconian measures should be prohibited. Emergency measures should not stand in the way of global trade and supply chain operations. On the other hand, countries should endeavor to enact trade facilitation policies. Measures like tariff reduction, the lifting of trade barriers, and unimpeded trade should be implemented proactively, whereas trade disputes such as trade wars and tariff wars should be avoided. In terms of pandemic prevention and economic growth, we should learn from China's experience and implement production by classification according to the costs and benefits of pandemic prevention and production in various industries. First of all, no matter how severe the pandemic situation is, it is necessary to ensure the production of enterprises supplying medical materials, water, electricity, gas, communications and other basic living materials. Basic material support is the basis of pandemic prevention and control, and only by ensuring the production of these enterprises can we better prevent and control the pandemic. Second, governments should ensure safe production among enterprises by category and in stages on the premise that prevention and control can be carried out effectively. Enterprises in which employees can be segregated during production, those where production and consumption are separable, and those that encounter less negative impact from pandemic prevention and control can carry out production with specific conditions during the outbreak. However, enterprises that are unable to meet these criteria must wait for the pandemic to ease or end before they can resume production.

To optimize incentive measures, international organizations should reward countries that actively engage in global pandemic governance. They should offer them incentives such as waiving or reducing the current year's membership fees and lowering import and export tariffs.

### Recommendations for Partaking in Cooperative Governance

The above discussion analyzed the influencing factors and enabling mechanisms associated with strategy realization for active cooperative governance. After strategy realization, measures to exploit each country's advantages to the fullest for the sake of global resource allocation against COVID-19 also warrant further investigation.

First of all, the COVAX Global New Crown Vaccine Initiative (New Crown Pneumonia Vaccine Implementation Program), jointly led by the World Health Organization and the GAVI Alliance, is currently the most effective mechanism for the equitable sharing of safe and effective vaccines worldwide, with the goal of promoting equitable global vaccine distribution. Effective vaccines should become global public goods, and first be provided to people in urgent need around the world (35). The ultimate victory in the global fight against the pandemic can only be achieved if countries around the world work together to ensure fair, equitable, and transparent distribution of the COVID-19 vaccine worldwide, and actively build a human health community (36). Moreover, relief resources including medical equipment, COVID-19 research and development, and medical personnel should be allocated inter-regionally according to the varying numbers of infections and severity of supply shortage among countries (37). Given the limited resources, the top priority should be to save as many lives as possible. With China being the world's largest manufacturer of medical protective wear and surgical masks (38), the Chinese government and enterprises have orchestrated multiple supply donations to European regions in a bid to overcome resource limitations. Second, as national supply reserves are limited in the time of closed-off management, the trade of daily essentials such as drugs and food must be sustained (39–41). Breaking the supply chain will jeopardize the domestic supplies of resources in the lesser endowed import countries. Should the pandemic show trends of extending into the long term, the exhaustion of domestic resources coupled with restricted imports will be a time bomb that can set off domestic crises. Furthermore, the excessive bans on aviation and transport have disrupted the order of the international trade system (42), crippling the role of resource-endowed countries in resource allocation. Third, as the global movement of the population will accelerate the spread of the virus, countries should make a coordinated effort to manage the movement of people (43, 44). While the hardest-hit countries should see it as their foremost task to restrict outbound travels, recovering countries should commit themselves to curtail inbound travels, while countries nearing the tail end of the outbreak should focus on blocking imported cases. Lastly, due to the devastating economic fallout of the pandemic, relief funds should be established with international cooperation to aid economically challenged countries. As poorer countries suffer the risks of higher incidence and mortality rates, the funds should serve the functions of reciprocal aid-giving and risk-sharing to expand the capacity of vulnerable regions in disease control and prevention.

## Limitation

Although this study scientifically expounds the view of international cooperation to respond to the COVID-19 by using game theory, it still has the following limitations. First, the game theory assumes that all subjects participating in decision-making are rational and represent their interests. However, in reality, the behavior of countries cannot keep rational all the time, which will affect the applicability of the conclusion. Secondly, although we consider the factors that affect the cooperation between countries as much as possible, there are still some factors that are difficult to consider, such as political factors, which will affect national decision-making. Third, our results have not taken into account the dynamic changes of the COVID-19. Despite the limitations, this study has important implications for international cooperation to manage pandemics.

## Conclusions

In the face of the global public health threats posed by the COVID-19 pandemic, active cooperative governance has evolved into the strongest weapon against the outbreak. That being said, some national governments have stuck to passive solutions due to an inadequate understanding of the dangers of the outbreak. To facilitate international cooperative governance, this paper studies the factors of active cooperative governance in each pandemic area based on the evolutionary game method, and based on the analysis of influencing factors, gives the policy path of how to promote international cooperation in a pandemic situation and how to carry out national cooperative governance in a pandemic situation.

What factors affect the international cooperation of pandemic control? First, if the costs of active pandemic governance, the economic losses incurred by such governance, and the costs of enabling the strategic choice of active cooperative governance are lower, the affected countries are more likely to choose the strategy of active cooperative governance over time. Second, if the individual returns of active pandemic governance, the public and shared returns of active cooperative governance, and the socio-economic impacts of the pandemic are higher, the affected countries are more likely to adopt the strategy of active cooperative governance over time. Third, if the coalition punishes countries for passive pandemic governance, subsidizes countries for active pandemic governance, and rewards alliances of active cooperative governance, the affected countries are more likely to opt for active cooperative governance over time.

The following recommendations are also proposed for enabling active cooperative governance among countries: (1) leveraging the role of international organizations to reduce the cost of realizing the strategy of active cooperative governance, (2) promoting the international exchange of related experiences to lower the cost of active pandemic governance, (3) sustaining productive and daily activities during the pandemic in a classified and hierarchical manner to reduce the economic losses incurred by active pandemic governance, and (4) optimizing the incentive measures of international organizations to guide countries and effectively facilitate the selection of active cooperative governance strategies. The marginal contribution of this study lies in drawing upon the evolutionary game perspective to identify the enabling mechanisms and cooperative pathways underlying international cooperative governance.

How to carry on the international pandemic situation cooperation governance? First, in terms of medical material management, global allocation of medical equipment resources, collective cooperation in scientific research, and relevant assistance from medical staff; second, in terms of supply management of living materials, global allocation of food and other necessities of life, and international trade cannot prohibit or restrict exports; third, in terms of population flow and cooperation management, countries in the severely affected areas should restrict population Outflow: countries with a good pandemic situation should restrict the inflow of population, and countries close to the end of the pandemic should strictly prevent the import from abroad. Fourthly, in terms of fund management for pandemic control, through the United Nations and other international organizations, a fund pool for pandemic control should be set up to increase financial assistance to the severely affected areas, vulnerable areas, and economically difficult areas.

## Data Availability Statement

The original contributions presented in the study are included in the article/supplementary material, further inquiries can be directed to the corresponding author/s.

## Author Contributions

HY and XZ: conceptualization. HY, SH, and YW: methodology. XZ, XL, and ZS: validation. HY, YW, and LX: formal analyses and investigation. HY, YW, XL, and XZ: writing—original draft preparation. All authors contributed to the article and approved the submitted version.

## Conflict of Interest

The authors declare that the research was conducted in the absence of any commercial or financial relationships that could be construed as a potential conflict of interest.

## Footnotes

^1^Data sources: https://voice.baidu.com/act/newpneumonia/newpneumonia/?from=osari_pc_3.
